# Comparison of Follicular Helper T-Cell Markers with the Expression of the Follicular Homing Marker CXCR5 in Peripheral T-Cell Lymphomas—A Reappraisal of Follicular Helper T-Cell Lymphomas

**DOI:** 10.3390/ijms25010428

**Published:** 2023-12-28

**Authors:** László Krenács, Dóra Krenács, Zita Borbényi, Erika Tóth, Anna Nagy, Klára Piukovics, Enikő Bagdi

**Affiliations:** 1 Laboratory of Tumor Pathology and Molecular Diagnostics, 6726 Szeged, Hungarybagdieniko67@gmail.com (E.B.); 2 Division of Haematology, Department of Internal Medicine, Albert Szent-Györgyi Clinical Center, University of Szeged, 6721 Szeged, Hungary; 3 Department of Pathology, National Institute of Oncology, 1122 Budapest, Hungary; toth.erika@oncol.hu; 4 1st Department of Pathology and Experimental Cancer Research, Faculty of Medicine, Semmelweis University, 1085 Budapest, Hungary

**Keywords:** nodal peripheral T-cell lymphoma, angioimmunoblastic type, follicular type, NOS type, follicular T helper cell phenotype, CD134, CXCR5, follicular dendritic cell meshwork

## Abstract

Peripheral T-cell lymphomas (PTCLs) expressing multiple follicular T helper (TFH) cell-related antigens are now classified as TFH lymphomas (TFHL), including angioimmunoblastic, follicular, and not otherwise specified (NOS) types. CXCR5 is the TFH cell-defining chemokine receptor that, together with its ligand CXCL13, plays a critical role in the development of follicles and the positioning of TFH and B cells within follicles. A comprehensive immunomorphologic study was performed to investigate the expression pattern of CXCR5 in a large cohort of nodal PTCLs, particularly those with a TFH cell phenotype, and to compare its expression with six other TFH cell-related antigens. We found that CXCR5 is widely expressed in neoplastic TFH cells, except in TFHL-NOS, and represents a specific marker of this lymphoma entity. Our results suggest that CXCR5 directs the distribution of neoplastic T cells in the affected lymph nodes and may influence the formation of the pathognomic pathological FDC network.

## 1. Introduction

The peripheral T-cell lymphomas (PTCL), malignant neoplasms of mature T cells, represent a rare and particularly diverse, clinically usually aggressive subgroup of lymphoid malignancies that arise in lymph nodes or extranodal sites [1,2,3]. Neoplastic cells in a significant subset of nodal PTCLs express multiple follicular T helper (TFH) cell-related antigens [1,2,3,4,5], and the latest lymphoma classification schemes assign these lymphomas to a single entity—TFH lymphoma (TFHL)—with 3 subtypes: angioimmunoblastic-type (TFHL-AI), follicular-type (TFHL-F), and not otherwise specified-type (TFHL-NOS) [2,3]. The TFHL-AI can be characterized by three histologic patterns based on the presence of hyperplastic B-cell follicles (TFHL-AI-1), partial or complete lymph node effacement with depleted B-cell follicles (TFHL-AI-2), and complete effacement with prominent FDC proliferation (TFHL-AI-3) [6]. The TFHL cases tend to be clinically aggressive malignancies affecting mostly elderly patients and characterized by an advanced-stage disease with generalized lymphadenopathy, hepatosplenomegaly, and complications due to immune dysregulation [1,2,3,7]. The prognosis is dismal, with a median survival of less than three years [7]. The progression of T-cell lymphoma, secondary aggressive B-cell lymphoma, or recurrent opportunistic infections can lead to a patient’s death [1,2,3,7].

The CXC chemokine receptor type 5 (CXCR5), a transmembrane protein, is the defining marker of TFH cells that enables them to migrate into the B-cell follicles [8,9,10,11]. Previously, we have shown that neoplastic T cells of angioimmunoblastic T-cell lymphoma (now TFHL-AI) express CXCR5 [4].

Aiming to explore the significance of CXCR5 in TFHLs, we performed a comprehensive immunomorphological study in a large cohort of PTCL cases and compared the expression of CXCR5 and six other TFH cell-related antigens.

## 2. Results

### 2.1. CXCR5 Staining Pattern in Non-Neoplastic and Neoplastic TFH Cells

**Figure 1:**
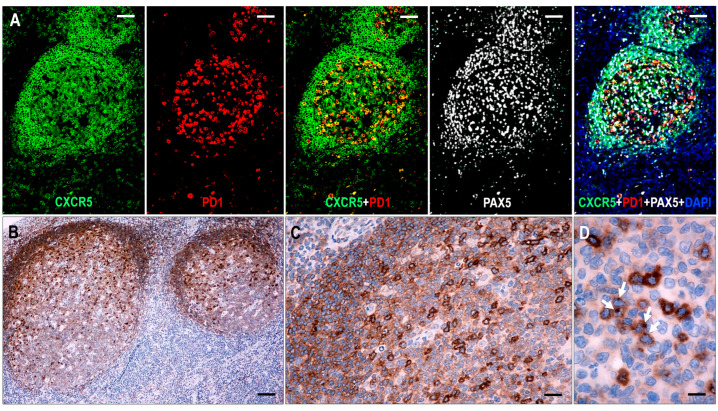
Expression of CXCR5 in hyperplastic follicles. (**A**) All follicular lymphoid cells, including GC B cells, mantle zone cells, and follicular T cells, show CXCR5 expression. In follicular T cells, CXCR5 is colocalized with PD1, whereas in B cells, it is colocalized with PAX5 (multiplex immunofluorescence labeling, ×100, scale bar = 100 μm). (**B**–**D**) The mantle zone cells demonstrate moderate staining, while the GC B cells have variable cytoplasmic CXCR5 staining, with moderate intensity in the light zone and weak intensity in the dark zone. The follicular T cells are presented with strong cytoplasmic CXCR5 staining with dot-like condensations in the Golgi region (white arrows). ((**B**) ×50, scale bar = 200 μm; (**C**) ×200, scale bar = 50 μm; (**D**) ×1000, scale bar = 10 μm).

In hyperplastic lymphoid tissues, TFH cells presented moderate to strong cytoplasmic CXCR5 staining with dot-like condensations in the Golgi region (Figure 1). All follicular B cells showed CXCR5 expression. The mantle zone cells exhibited moderate to strong diffuse cytoplasmic staining, while the germinal center (GC) B cells had variable cytoplasmic CXCR5 staining, with moderate intensity in the light zone and weak intensity in the dark zone. In addition, marginal zone B cells of hyperplastic tonsils showed moderate CXCR5 expression.

In most lymphoma cases, the abnormal TFH cells showed a dot-like staining in the Golgi region with or without diffuse cytoplasmic staining. In cases where CXCR5 gave only polar dot-like staining, the number of positive lesional T cells appeared to be lower than with markers that produce circumferential membrane staining, such as PD1 and ICOS. Diffuse cytoplasmic CXCR5 positivity of moderate intensity was a consistent finding in residual mantle zone B cells, which was also used as an endogenous positive control for the CXCR5 immunostaining. Extrafollicular B immunoblasts (B-IBs) and Hodgkin-Reed-Sternberg-like (HRS-like) cells showed dot-like Golgi plus variable membrane-bound staining, which was, in most instances, easily distinguishable from the staining pattern of the abnormal TFH cells.

### 2.2. Determination of TFH Cell Phenotype in PTCL Cases

**Figure 2:**
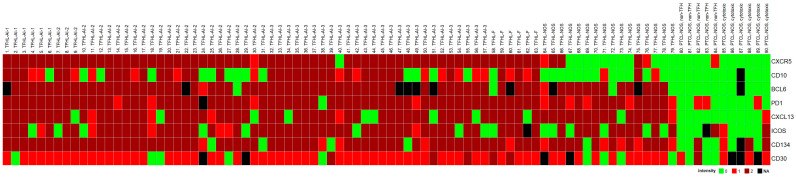
Heatmap of TFH cell marker expression in PTCL cases studied. The TFHL cases express most of the TFH cell markers, with the exception of TFHL-NOS, where CXCR5 and CD10 are mostly negative.

Seventy-nine cases, all of which were CD4+, had at least four of the seven TFH cell markers, indicating TFHL. Eighty-nine percent of TFHL cases (70/79) had five or more TFH cell antigens expressed in the neoplastic cells (Figure 2). Positivity rates of TFH cell markers ranged from 63% to 99%. PD1, BCL6, and CD134 had the highest sensitivity of 99% (77/78 cases), 94% (67/71 cases), and 94% (74/79 cases), respectively, while CD10 had the lowest sensitivity of 63% (50/79 cases). CXCR5 had a positivity rate of 85% (67/79) in the entire TFHL group but showed one of the highest sensitivities of 98% (62/63 cases) in the TFHL-F plus TFHL-AI cohort. Overall, BCL6 showed one of the highest sensitivities, in addition to the highest specificity and accuracy (Table 1).

All 11 non-TFH type PTCL NOS cases expressed fewer than four TFH cell-related antigens. Four of them were CD4+, four cases were CD8+, and three cases showed a CD4/CD8 double-negative phenotype. Two CD4+ non-cytotoxic and one CD4+ cytotoxic cases expressed at least two TFH cell markers but were negative for the remaining five markers and were classified as PTCL NOS of non-TFH cell type. One CD8+/TIA-1+ cytotoxic PTCL NOS case expressed three TFH-cell-related markers (CXCL13, ICOS, and CD134). One CD8+ and two CD4/CD8 double-negative cytotoxic PTCL NOS cases expressed at least one TFH cell-related antigen (PD1 or CD134). CXCR5+ neoplastic T cells were found in one of the CD4+ cases (1/11, 9%), with a membranous staining pattern distinct from the Golgi staining seen in the vast majority of TFHL. CD10 and BCL6 showed no positive abnormal cells (both 0/10) in non-TFH type PTCL NOS. Here, 1/11 (9%) cases were CXCL13+, 3/11 (27%) were PD1+, 3/10 (30%) were ICOS+, and 4/10 (40%) were CD134+.

The CXCR5 expression showed a statistically significant correlation with the TFH cell phenotype compared to the non-TFH-PTCL cases (Mann–Whitney U-test: *p*-value < 0.0001; z-score 4.44084).

### 2.3. Immunomorphology of TFHL

The immunomorphological characteristics are summarized in Table 2.

#### 2.3.1. TFHL-AI-1

**Figure 3:**
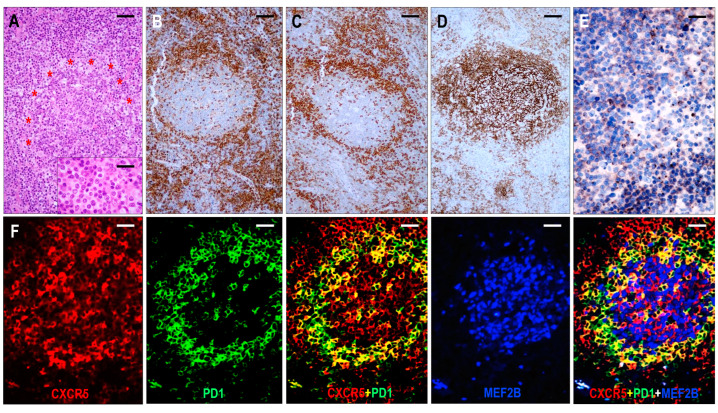
Immunomorphology of TFHL-AI-1. (**A**) HE-stained section shows a hyperplastic follicle with retained GC and a ring-like accumulation of abnormal cells (red asterisks). The mantle zone is inconspicuous (×100, scale bar = 100 μm). Inset: Small abnormal cells have pale cytoplasm (×1000, scale bar = 25 μm). The abnormal cells accumulate at the periphery of GC showing CD4 (**B**) and PD1 (**C**) positivity (both ×50, scale bar = 200 μm). (**D**) CD21 highlights dense, nonpolarized FDC meshwork (×50, scale bar = 200 μm). (**E**) Abnormal T cells exhibit dot-like cytoplasmic CXCR5 staining (×400, scale bar = 25 μm). (**F**) CXCR5 is expressed in an abnormal follicle and is colocalized with PD1 in neoplastic TFH cells and with MEF2B in residual GC B cells (multiple immunofluorescence labeling, ×200, scale bar = 50 μm).

Six cases were consistent with pattern 1 TFHL-AI, showing follicular hyperplasia-like features with indistinct or distorted mantle zones and an often ring-like accumulation of abnormal T cells in the periphery of distorted hyperplastic germinal centers (GCs) (Figure 3). High endothelial venules (HEVs) showed variable proliferation. Occasionally, HEVs that penetrated radially into the GCs or were located within the tumor cell ring were observed.

The neoplastic cells were presented as small to medium-sized CD3+ or CD5+ and CD4+ atypical T cells with pale cytoplasm, expressing at least 6 of the 7 TFH markers (Figure 3). All 6 cases were CXCR5+/BCL6+/PD1+/CXCL13+/ICOS+/CD134+, while 5/6 cases (83%) were CD10+. In addition, a considerable proportion of the abnormal TFH cells displayed weak to moderate CD30 positivity in each case. A follow-up biopsy was available in 3 cases, including 2 cases with persistent TFHL-AI-1 and 1 case with progression to TFHL-NOS with CXCR5-negative neoplastic cells.

**Figure 4:**
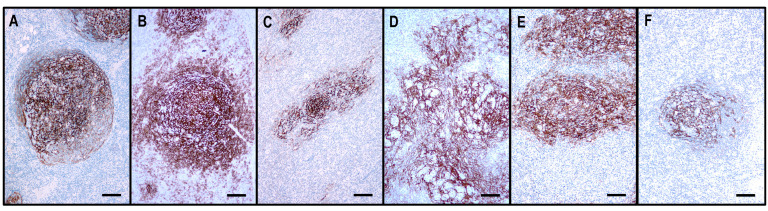
The architecture of the CD21+ FDC meshwork in reactive hyperplasia and TFHLs. (**A**) The hyperplastic follicle shows a polarized FDC arrangement with a dense meshwork in the light zone and loosely arranged projections in the dark zone. (**B**) In TFHL-AI-1, the FDC meshwork is nonpolarized, dense, and slightly expanded. (**C**) In TFHL-AI-2, the FDC meshwork is typically regressed with irregular borders. (**D**) In TFHL-AI-3, the FDC meshwork demonstrates an enormous expansion with a widely confluent, interconnected pattern. (**E**) TFHL-F has a dense, irregularly expanded, focally confluent FDC meshwork. (**F**) In the TFHL-NOS with a T-zone pattern, the FDC meshwork is atrophic and shows a largely intact organoid pattern. (All CD21, ×100, scale bars = 100 μm).

Follicular dendritic cells (FDCs), highlighted by CD21, CD23, and CXCL13, formed a slightly disarranged, nonpolarized, and marginally expanded FDC meshwork (Figure 4). The overwhelming majority of the neoplastic TFH cells appeared within the follicular compartment, i.e., within the FDC network, at the outer boundary of the hyperplastic GCs (Figure 3); however, scattered extrafollicular/paracortical TFH antigen-positive atypical T cells were also noted in each case.

The GCs were at least partially preserved and consisted of MEF-2B+ B cells (Figure 3), mostly with centroblastic morphology, and were surrounded by disarranged CXCR5+/IgD+ mantle zones. GC B cells also showed CXCR5 positivity. In addition, primary follicle-like CXCR5+/IgD+ small B-cell aggregates were also seen, often pressed against the subcapsular sinuses. The paracortical compartment appeared polymorphic, with a few B-IBs and HRS-like cells.

All 5 cases tested for LMP1 expression and EBER in situ hybridization demonstrated from 1% to focally up to 30% EBV-positive cells with a cytomorphological spectrum from small plasmacytoid cells to HRS-like cells. A proportion of GC B cells and the majority of the extrafollicular B-IBs and HRS-like cells showed moderate to strong CD30 positivity, unrelated to EBV content.

#### 2.3.2. TFHL-AI-2

Here, 25 cases corresponded to TFHL-AI-2. These cases exhibited a complete disarrangement in the lymph node architecture, showing regressed B-cell-depleted follicles with irregular FDC networks and a polymorphic paracortical infiltrate with mild to moderate HEV proliferation.

The neoplastic cells were CD3+ and CD4+ medium- to large-sized pathological T cells with pale cytoplasm that expressed at least 5 of the 7 TFH cell markers (Figure 2). All cases (25/25, 100%) exhibited CXCR5+ neoplastic T cells. The expression rates of the other TFH cell antigens were as follows: PD1+ 24/24 (100%), BCL6+ 23/24 (96%), ICOS+ 21/25 (84%), CD134+ 23/25 (92%), CXCL13+ 22/25 (88%), and CD10+ 15/25 (60%). In addition, the abnormal TFH cells showed almost consistent (20/24 cases, 83%) but weak CD30 positivity. A follow-up biopsy was available in 5 cases, including 3 cases with persistent TFHL-AI-2, 1 case with progression to TFHL-AI-3, and 1 case with progression to TFHL-NOS with CXCR5-negative neoplastic cells.

**Figure 5:**
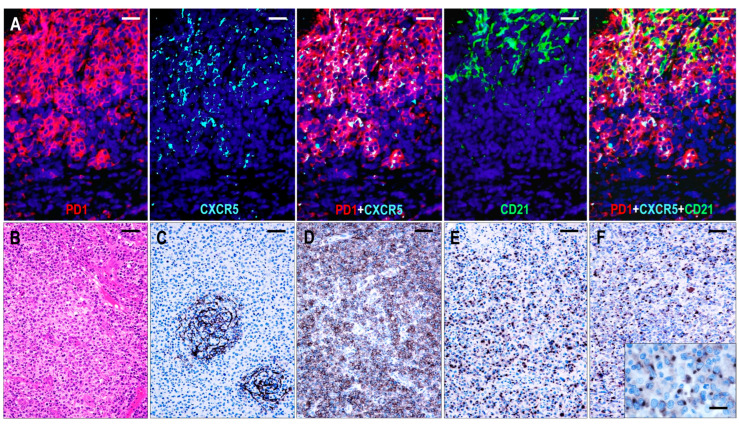
Immunomorphology of TFHL-AI-2. (**A**) In a type A TFHL-AI-2 case, abnormal TFH cells, expressing both PD1 and CXCR5, accumulate within and around the CD21+ FDC meshwork (multiple immunofluorescence labeling with DAPI nuclear staining, ×400, scale bar = 25 μm). (**B**) Type B TFHL-AI-2 case shows a typical polymorphic paracortical infiltration (HE stain, ×200, scale bar = 50 μm). The neoplastic T cells are localized predominantly in the paracortical region, outside of the CD21+ regressed FDC network (**C**), and express PD1 (**D**), CXCL13 (**E**), and CXCR5 (**F**). Inset highlights dot-like cytoplasmic CXCR5 positivity of neoplastic T cells. (C–F, ×100, scale bars = 100 μm; inset ×1000, scale bar = 25 μm).

The FDC meshwork was mostly atrophic, with irregular margins. Focal expansion with a minimally confluent pattern and regressed follicles with a concentric FDC arrangement were typical findings (Figure 4). Based on the distribution of abnormal TFH cells, the TFHL-AI-2 cases could be divided into two subgroups. In 8 cases, the abnormal TFH cells were mainly confined to the FDC meshwork or the adjacent perifollicular area, while significantly fewer cells were found in the paracortical compartment (TFHL-AI-2 type A) (Figure 5). In the remaining 17 cases, however, the abnormal TFH cells occurred predominantly in the extrafollicular region, and a minor subset was found within the regressed FDC meshwork (TFHL-AI-2 type B) (Figure 5).

The follicles had scarce or no MEF2B+ GC B cells. Almost without exception, sheets with displaced small B cells of the CXCR5+/IgD+ mantle zone-type small B cells were found, often pushed to the periphery of the lymph nodes. A variable number of extrafollicular B-IBs and HRS-like cells were present in almost all cases, and these cells were found to be CXCR5+ in 80% (20/25) of the cases. B-IBs and HRS-like cells consistently showed moderate to strong CD30 membrane positivity, which was not related to the EBV content.

When tested for LMP1 expression and EBER in situ hybridization, EBV-positive cells were found in 80% (20/25) of the cases. Scattered EBV-positive B cells, small lymphocytes, plasma cells, B-IBs, and occasional HRS-like cells were detected in most cases; however, focally up to 30% of EBV-containing cells, including many HRS-like cells, were observed in 3 cases. Of the 20 cases having CXCR5+ HRS-like cells, 12 cases (60%) showed EBV-positive HRS-like cells.

Furthermore, 11 of the 25 TFHL-AI-2 cases (44%) were associated with a variety of B-cell proliferations, which included polyclonal plasmacytosis (1 case), EBV-positive mucocutaneous ulcer (1 case), EBV-positive polymorphic B-cell proliferation (4 cases), EBV-negative polyclonal large B-cell proliferation (2 cases), EBV-negative diffuse large B-cell lymphoma (DLBCL) (2 cases), and EBV-positive gastric DLBCL (1 case).

#### 2.3.3. TFHL-AI-3

**Figure 6:**
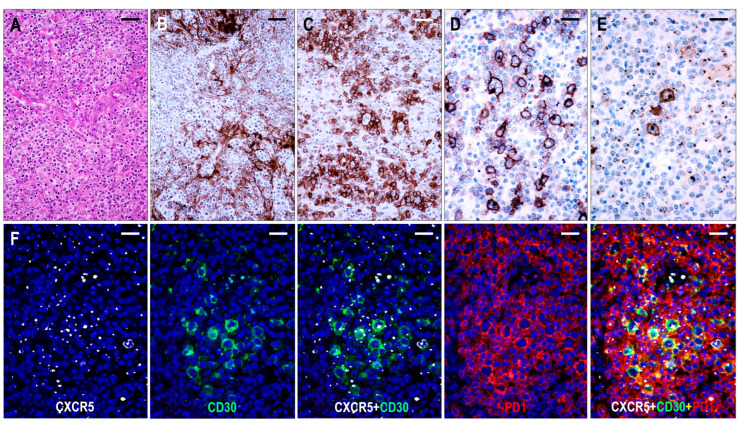
Immunomorphology of TFHL-AI-3. (**A**) HE-stained section demonstrates the characteristic polymorphic infiltration with HEV proliferation and atypical cells of pale cytoplasm (×200, scale bar = 50 μm). (**B**) CD21 expression highlights the greatly expanded, confluent abnormal FDC meshwork (×200, scale bar = 50 μm). (**C**) CD134+ neoplastic T-cells surround HRS-like cells (×200, scale bar = 50 μm). (**D**) Strongly CD30+ large B cells and weakly CD30+ neoplastic T cells in the background are seen (×400, scale bar = 25 μm). (**E**) HRS-like cells showing CXCR5 positivity with strong cytoplasmic and membrane staining surrounded by neoplastic T cells with typical dot-like CXCR5 positivity are demonstrated (×400, scale bar = 25 μm). (**F**) The CXCR5 is coexpressed with CD30 in the neoplastic T cells and HRS-like cells and with PD1 in the neoplastic T cells (multiple immunofluorescence labeling with DAPI nuclear staining, ×400, scale bars = 25 μm).

Here, 26 cases were consistent with TFHL-AI-3. These cases were characterized by a complete effacement of the lymph node architecture, displaying markedly abnormal FDC networks, and a polymorphic infiltrate with prominent arborizing HEV proliferation (Figure 6).

The neoplastic cells appeared as CD3+ and CD4+ medium- to large-sized T cells with pale cytoplasm, usually forming small clusters and expressing at least 5 of the 7 (4/6 in 1 case) TFH cell markers (Figure 2). All but one case (25/26, 96%) demonstrated CXCR5 expression in the neoplastic T cells. The incidence of the other TFH cell markers was as follows: PD1+ 25/26 (96%), CXCL13+ 21/26 (81%), CD134+ 24/26 (92%), ICOS+ 23/26 (88%), BCL6+ 22/22 (100%), and CD10+ 22/26 (85%). In addition, the neoplastic cells displayed weak to moderate CD30 positivity in most cases (24/25, 96%) (Figure 6). Significant numbers of neoplastic TFH cells occurred in both the intrafollicular (i.e., within an abnormal FDC network) and extrafollicular compartments; however, intrafollicular localization was prevalent in 6 cases. A follow-up biopsy was available in 1 case showing persistent TFHL-AI-3 with increasing tumor cell content.

FDCs displayed an enormous expansion with widely confluent meshwork (Figure 4 and Figure 6). In many cases, HEVs were entrapped by the expanded, interconnected FDC meshwork. The FDC meshwork held no residual GC B cells. Here, 10–30% mantle zone-type CD20+/CXCR5+/IgD+ small B-cells, occurring in haphazardly distributed sheets next to the FDC meshwork or confining to the periphery of the affected lymph nodes, were almost invariably found. In one case, the small B cells formed disorganized confluent sheets and represented more than 50% of the lymphoid cells. Further, 1–10% of B-IBs and HRS-like cells were present in almost all (23/26, 88%) cases, which were frequently rosetted by the abnormal TFH cells (Figure 6). The B-IBs and HRS-like cells were found to be CXCR5+ in 17/23 (74%) cases (Figure 6). B-IBs and HRS-like cells consistently showed moderate to strong CD30 membrane positivity, which did not parallel EBV content.

The presence of EBV was detected in all but 2 cases (23/26, 88%). The number of EBV-positive cells varied from 1-20% of lymphoid cells and ranged from small lymphocytes to HRS-like cells. Twelve of the 17 cases (71%) having CXCR5+ B-IBs and HRS-like cells showed concomitance of EBV and CXCR5.

Five cases demonstrated various B-cell proliferations, including CXCR5+/IgD+ small B-cell hyperplasia (1 case), polyclonal plasmacytosis (1 case), EBV-negative polymorphic B-cell proliferation with kappa light chain restriction (1 case), EBV-negative polymorphic polyclonal proliferation (1 case), and EBV-positive polymorphic polyclonal B-cell proliferation (1 case).

#### 2.3.4. TFHL-F

**Figure 7:**
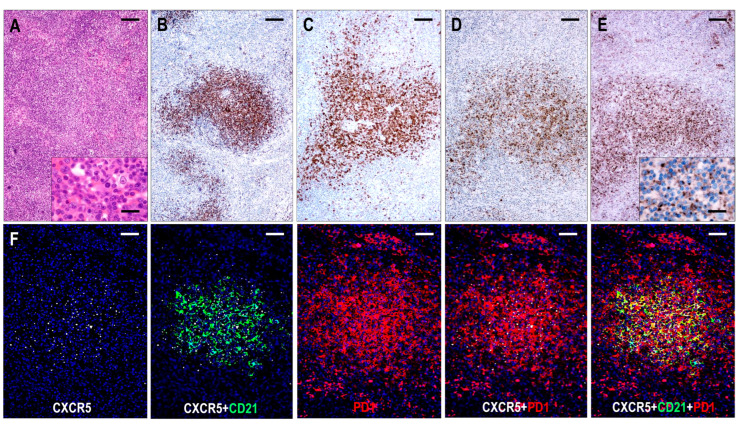
Immunomorphology of TFHL-F. (**A**) The abnormal infiltrate is characterized by vague nodularity in the H&E-stained section (×50, scale bar = 200 μm). In the inset, the lesional abnormal cells show minimal atypia (×1000, scale bar = 25 μm). The abnormal cells form nodules while expressing CD4+ (**B**), CD10+ (**C**), CXCL13+ (**D**), and CXCR5+ (**E**). (B–E, ×50, scale bars = 200 Mm). Inset highlights dot-like CXCR5 staining in the abnormal T cells (×1000, scale bar = 25 μm). (**F**) The abnormal T-cell aggregates are CXCR5+, localized within the FDC meshwork, and coexpress PD1 (multiple immunofluorescence labeling, DAPI nuclear staining, ×100, scale bars = 100 μm).

Six cases were compatible with TFHL-F, showing multiple follicle-like irregular nodules mostly composed of CD3+ and CD4+ small- to medium-sized abnormal T cells with pale cytoplasm, which expressed at least 4/7 TFH cell markers (Figure 2). Neoplastic T cells were CXCR5+ in 6/6 (100%), PD1+ in 6/6 (100%), CXCL13+ in 5/6 (83%), CD134+ in 6/6 (100%), ICOS+ in 4/5 (80%), BCL6+ in 4/5 (80%), and CD10+ in 5/6 (83%) cases. Moreover, neoplastic cells displayed a consistent (6/6, 100%) CD30 expression with a variable, typically weak intensity. Neoplastic TFH cell aggregates are situated within dense, irregularly expanded, focally confluent CD21+ or CD23+ and CXCL13+ FDC meshwork (Figure 4 and Figure 7). The extrafollicular compartment represented a disrupted paracortical tissue with scattered atypical TFH cells. A follow-up biopsy was available in 3 TFHL-F cases, and progression into TFHL-AI-2 was noticed in 2 cases.

The T-cell-rich neoplastic aggregates held no MEF2B+ GC B cells but scattered (up to 10%) CXCR5+/CD30+ B-IBs and a few HRS-like cells. Sheets of dislocated CD20+/CXCR5+/IgD+ mantle zone-type small B cells adjacent to the neoplastic T-cell nodules or pushed to the subcapsular region were observed. In one case, these small B cells were composed of 50% of the lymphoid cells and formed irregular primary follicle-like aggregates that were mixed with large sheets of intrafollicular neoplastic T-cells.

EBV-positive B cells were present in all (6/6, 100%) cases, with a frequency of 1–10% of lymphoid cells, ranging from small lymphocytes to HRS-like cells. EBV-positive large B cells and HRS-like cells were predominantly located within the follicular compartment.

#### 2.3.5. TFHL-NOS

Sixteen cases were found to be TFHL-NOS. These cases showed complete effacement of the lymph node architecture. The neoplastic T cells were CD3+ and CD4+ small- to large-sized, often with pale cytoplasm, exclusively situated in the extrafollicular/paracortical compartment, and expressed not less than 4/7 TFH cell markers (Figure 2). These T cells were CXCR5+ in 5/16 (42%), PD1+ in 16/16 (100%), CXCL13+ in 15/16 (94%), CD134+ in 15/16 (94%), ICOS+ in 11/16 (69%), BCL6+ in 12/14 (86%), and CD10+ in 3/16 (19%) cases. Additionally, most neoplastic T cells showed a variable, typically weak CD30 positivity in 11/14 cases (79%).

**Figure 8:**
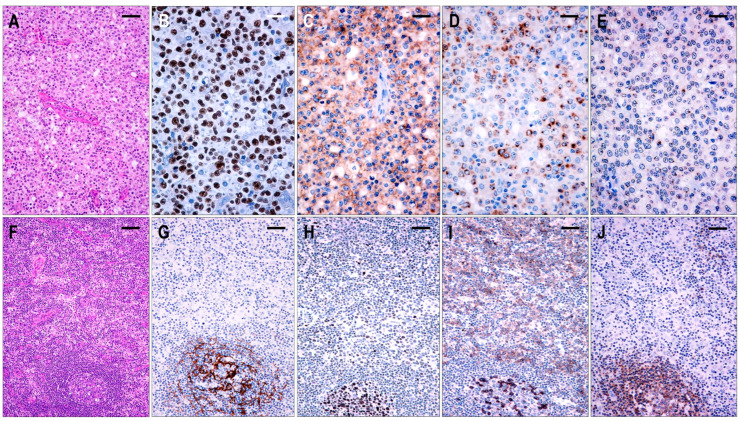
Immunomorphological characteristics of TFHL-NOS. (**A**) Type A TFHL-NOS case shows a monomorphic infiltrate of medium-sized atypical cells with pale cytoplasm (H&E, ×200, scale bar = 50 μm). The neoplastic T cells are EBER+ (**B**) and coexpress PD1 (**C**), CXCL13 (**D**), and CXCR5 (**E**) (B–E, ×400, scale bars = 25 µm). ((**F**) Type B TFHL-NOS shows polymorphic paracortical infiltration (H&E ×50, scale bar = 200 µm). (**G**) An uninvolved residual atrophic follicle with CD21+ FDC meshwork is highlighted (×100, scale bar = 100 µm). The neoplastic T cells are BCL6+ (**H**) and PD1+ (**I**) but lack CXCR5 (**J**). Note the intrafollicular non-neoplastic CXCR5+ T and B cells. (H–J, ×100, scale bars = 100 µm).

Based on the architectural alterations, cytomorphology, and tumor cell content, the TFHL-NOS cases could be subdivided into three subgroups. Three cases with a rather monomorphic infiltration of CXCR5+ medium to large neoplastic T cells without traces of follicular structures represented TFHL-NOS type A (Figure 8). Five cases showed polymorphic paracortical infiltration and uninvolved residual atrophic follicles (consistent with a T-zone lymphoma pattern), and the neoplastic T cells lacked CXCR5, corresponding to type B of TFHL-NOS (Figure 8). The atrophic follicles contained MEF2B+ GC B cells, tingible body macrophages, and a small number of non-neoplastic TFH cells. Finally, classic polymorphic infiltrate and HEV proliferation were found in 8 cases, which demonstrated only delicate traces or absence of FDC meshwork, corresponding to type B of TFHL-NOS type C, and only 2 of them had CXCR5+ neoplastic cells.

The overall difference between CXCR5 expression in TFHL-NOS and other TFHL subtypes was found to be statistically significant (Mann-Whitney U-test: *p*-value < 0.0001; z-score 4.849).

Here, 5–20% mantle zone-type CD20+/CXCR5+/IgD+ small B-cells were seen in most cases. Type B TFHL-NOS cases revealed an atrophic but almost intact mantle zone surrounding depleted but involved GCs, while, in the other cases, disorganized, haphazardly distributed small B cell aggregates appeared. One to 10% of CD30+ B-IBs and HRS-like cells were present in 11/16 (69%) cases, of which 8/11 (73%) cases displayed CXCR5+ B-IBs and HRS-like cells.

EBV was detected in 10/16 (63%) cases. In one type A case, the neoplastic large T cells showed homogenous EBV positivity, while, in the remaining cases, B cells were EBV-positive with a frequency of 1–20% of all lymphoid cells, ranging from small lymphocytes to HRS-like cells. Nine cases had CXCR5+ B-IBs and HRS-like cells that showed no association with the presence of EBV.

Abnormal B-cell proliferations were observed in 3 cases, including polyclonal plasmacytosis (2 cases) and EBV+ polymorphic B-cell proliferation (1 case).

## 3. Discussion

PTCLs are uncommon neoplasms of mature T cells with a usually aggressive course [1,2,3]. In a significant subset of PTCLs, the neoplastic T cells show a TFH cell phenotype and are considered the neoplastic counterpart of normal TFH cells [1,2,3,4,5]. In current classification schemes, the TFH cell phenotype is defined by the presence of at least 2 or preferably 3 of the most frequently used TFH markers, PD1, CD10, BCL6, CXCL13, and ICOS, in addition to CD4 [2,3,12]. In this study, we performed a comprehensive TFH phenotyping in PTCLs with an extended 7-TFH marker panel by adding CXCR5 and CD134, which we had previously proven to be consistently expressed in the neoplastic T cells of TFHL-AI (formerly angioimmunoblastic T-cell lymphoma) [4]. We found that all TFHL cases had at least 4 of the 7 TFH cell-related markers in the neoplastic cells, and the vast majority expressed 5 or more such antigens. Interestingly, BCL6 proved to be the most specific and one of the most sensitive TFH cell markers. The use of MEF2B can facilitate this, as it allows us to easily separate BCL6+ GC B cells from BCL6+ TFH cells [13]. CD134 proved to be a suitable marker in this series, showing conspicuous membrane staining with comparable sensitivity and specificity to PD1, one of the most basic markers for TFH cells [14,15]. The CD134 is essential for activated T helper cell migration into B-cell follicles and cooperates with ICOS in maintaining the TFH cell phenotype [16,17]. Our results suggest that it could be a useful addition to TFH cell phenotyping.

One of our main goals with this study was to explore the expression pattern of CXCR5 in a large cohort of nodal PTCLs, particularly with a TFH cell phenotype. We found that the neoplastic T cells in the TFHL cases, apart from the TFHL-NOS, are CXCR5+. Since CXCR5 plays a key role in the migration of T and B cells into lymphoid follicles [8,9,11,18,19,20], we paid special attention to the distribution of neoplastic TFH cells. In the TFHL-AI-1, TFHL-AI-3, and TFHL-F types, CXCR5+ neoplastic T cells were predominantly localized in the diseased FDC meshwork, which is consistent with the follicular microenvironment, whereas the TFHL-AI-2 cases showed intermediate features. In TFHL-AI-2 type A cases, the neoplastic TFH cells were largely detected within or around the follicular compartment. In TFHL-AI-2 type B cases, the abnormal TFH cells were predominantly located in the paracortical compartment, with a minor intrafollicular subset. Nevertheless, both types of TFHL-AI-2 showed irregular, at least focally expanded FDC meshwork and some degree of intrafollicular involvement. Only one-third of the TFHL-NOS cases were CXCR5+, including all 3 cases with diffuse large T-cell lymphoma pattern (type A) and 2 polymorphic cases without FDCs (type C). However, in most TFHL-NOS cases, the neoplastic T cells lacked CXCR5 and accumulated in the paracortical compartment. These CXCR5-negative cases showed either an absent follicular structure or no abnormal T cells within the residual follicles, which later feature is consistent with the so-called T-zone lymphoma pattern. Previously, CXCR5-negative TFH-like phenotype has been designated as a primary cutaneous CD4+ small/medium T-cell lymphoproliferative disorder, suggesting a derivation from a distinct T helper-cell population that provides help to B cells outside the follicles [21,22,23,24]. Recently, unique CD4+ T cells were described in chronically inflamed tissues that provide help to B cells. They share many features with TFH cells, including expression of PD1, CXCL13, and ICOS, and they often lack CXCR5 [25,26,27]. These cells, however, occur in tertiary lymphoid structures; therefore, they cannot serve as a non-neoplastic counterpart of nodal TFHL-NOS. It seems much more likely that the neoplastic cells are defective TFH cells that cannot migrate into the follicles in the absence of CXCR5. Although, in some TFHL-NOS cases, CXCR5 was detectable in the neoplastic cells, follicular involvement was similarly absent. In these as well as in TFHL-AI-2 cases with predominantly extrafollicular involvement, a functionally defective CXCR5 molecule or dysregulated migratory machinery may be the explanation. Circulating CXCR5+ T helper cells in the blood, representing memory TFH cells and mature TFH cells located in the B-cell follicles of secondary lymphoid follicles, have been characterized [10,19,28,29], but the spatiotemporal determinants of migration in lymph nodes and other secondary lymphoid organs, from the HEV passage to the follicular entry, have not been fully elucidated [11,29,30,31]. CXCR5 is necessary but not sufficient for the follicular homing of TFH cells. CXCR5+ circulating T cells require a high expression of CCR7 to enter the lymph nodes, while upregulation of CXCR5 and downregulation of CCR7 are crucial for entering the follicle [10,32]. Dysregulation of this process may also contribute to neoplastic TFH cells being trapped in the extrafollicular compartment. Since CXCR5, in cooperation with its ligand CXCL13, plays a crucial role in the establishment of follicles, positioning of TFH and B cells in the follicles, and maintenance of the follicular microenvironment [8,9,19,33,34], our results suggest that CXCR5 directs the distribution of neoplastic T cells in the affected lymph nodes and contributes to the formation of the pathological FDC network.

TFH cells are essential for the functional differentiation of mature B cells, including the formation of GCs, affinity maturation of GC B cells, production of high-affinity antibodies, and generation of memory B cells [9,10,19,30,32]. All these functions can be impaired by the neoplastic transformation of the TFH cells. In addition to neoplastic T-cell proliferation, the TFHL cases examined showed a variety of alterations in the B-cell compartment. Depending on the type and probably biological stage of the disease, these alterations included hyperplastic or depleted B-cell follicles, a redistribution of mantle zone small B lymphocytes, and the appearance of large B cells with immunoblastic or sometimes HRS-like cytomorphology. In the early phase of the neoplastic process, the TFH cell function may be largely preserved, leading to the activation of GC-B cells and hyperplastic follicles, as seen in TFHL-AI-1. In certain cases, the neoplastic TFH cells accumulate in the follicles but lose the ability to attract B cells to form GCs, which could be the explanation for TFHL-F. As TFH cell function deteriorates, the number of GC-B cells gradually decreases, leading to follicular regression, which is the typical pattern for TFHL-AI-2. In the most advanced cases, the follicular compartment shows radical transformation with an excessive expansion of the FDC meshwork, which is seen in TFHL-AI-3. We found that the number of MEF2B+ GC B cells was inversely correlated with the growth pattern, being high in TFHL-AI-1 and completely absent in TFHL-AI-3. CD30+ activated large B cells with immunoblastic or rarely HRS-like morphology occurred in all TFHL types. In TFHL-F and -AI-3, most of these cells occurred in the follicular compartment, whereas in the other types, they were mainly found in the paracortical compartment and were often surrounded by neoplastic TFH cells in the tumor cell-rich areas. This pattern mimics Hodgkin’s lymphoma and can be misleading. It essentially represents the reverse image of Hodgkin’s lymphoma, having non-neoplastic large B cells in the center surrounded by the apparently atypical neoplastic TFH cells. The fact that the abnormal T cells are in direct contact with the CD30+ large B cells, forming rosettes around them, proves that these B cells attract and support the neoplastic TFH cells in the absence of GC B cells [35].

CXCR5 is expressed in both TFH cells and mature B cells, allowing them to migrate into B cell follicles [8,18,19,36,37]. Previous studies on mouse and human lymphoid tissues have shown that CXCR5 is mainly expressed in the light zone of the GC [20,36,38]. In this study, we also evaluated CXCR5 in hyperplastic human lymphoid tissues and established that, in addition to TFH cells, all follicular B cells also exhibited CXCR5 expression, including the mantle zone B cells with strong positivity, the GC light zone B cells with moderate positivity, and the GC dark zone B cells with weak positivity. The intensity parallels the polarity of the GC and can be explained by the density of the CXCL13-bearing FDC meshwork, which is denser in the light zone than in the dark zone. In TFHL cases with better preserved follicular structures, nearly intact CXCR5+/IgD+ mantle zones were found, while the advanced cases showed irregular collections of mantle zone-type small B cells adjacent to the abnormal FDC meshwork or pressed up against the subcapsular sinuses. The CD30+ large B cells, including B-IBs and HRS-like cells, frequently showed CXCR5 positivity; nevertheless, no relationship was found between the presence of EBV and CXCR5 expression. A similar rate of CXCR5 expression was described in HRS cells in classical Hodgkin’s lymphoma [38]. This observation proves an additional overlapping feature between TFHL and classical Hodgkin’s lymphoma. Further studies are needed to clarify the significance of CXCR5 expression in the non-GC large B cells of TFHL.

Lymph nodes affected by TFHL typically reveal a polymorphic infiltrate with extensive cellular and structural changes, including the proliferation of FDCs and HEVs [1,2,3,6]. Our results suggest that the FDC networks in all TFHL subtypes, except TFHL-NOS, are not simply residual skeletons of depleted B-cell follicles but may actively contribute to the pathologic process in collaboration with CXCR5, and virtually all types of TFHL show some degree of FDC alteration. The structure of the FDC networks is almost always affected and shows some distortion, while the full-blown cases reveal characteristically striking expansion. The density of the FDC meshwork may be similar to or even greater than that of the light zones of hyperplastic GCs. The diseased FDCs may either originate from pre-existing follicles or represent newly formed cells from fibroblastic reticular cells surrounding the HEVs [39,40]. This may explain why the pathologic FDC networks extend beyond the boundaries of normal follicles and why HEVs entrapped by the FDCs are so often observed.

## 4. Materials and Methods

### 4.1. Patients and Tissue Samples

Formalin-fixed and paraffin-embedded tissue samples of 90 PTCL cases were retrieved from the Laboratory of Tumor Pathology and Molecular Diagnostics, Szeged, Hungary, and the Department of Pathology, National Institute of Oncology, Budapest, Hungary. The collected cases were diagnosed on the basis of clinical information, histomorphology, immunophenotypic characteristics, and PCR clonality analysis for TCR-gamma and IgH gene rearrangements. Inclusion criteria were the availability of representative paraffin blocks or adequate unstained slides for additional immunohistochemical analysis. LK, DK, and EB revised and reclassified all cases based on the expanded TFH phenotyping data and according to the current classification schemes [2,3].

The demographic and pathological data of the analyzed cases are summarized in Supplementary Table S1.

Our series included 79 cases of TFHL and 11 non-TFH-cell PTCL NOS cases, including 6 cases with cytotoxic phenotypes. The 79 TFHL cases were composed of 57 TFHL-AI, 6 TFHL-F, and 16 TFHL-NOS cases (Supplementary Materials Table S1). Overall, 133 formalin-fixed and paraffin-embedded tissue samples were evaluated.

Clonal TCR rearrangement was proven in 62/68 cases (91%). In addition, 7/35 cases (20%) showed clonal IgH gene rearrangement.

This retrospective study was approved by the ethics committee of the University of Szeged (reference no. 4798/2020) and conducted in accordance with the Declaration of Helsinki. 

### 4.2. Immunohistochemistry and Evaluation

Immunohistochemical reactions were performed on formalin-fixed and paraffin-embedded samples. Briefly, 2-μm-thick paraffin sections were routinely deparaffinized and heat-treated with appropriate antigen retrieval buffer solutions using a household electronic pressure cooker. After protein blocking (RE7102, (Leica/Novocastra, Deer Park, IL, USA), sections were incubated with primary antibodies at room temperature for 60 min. Detection was performed using a Novolink polymer kit (Leica/Novocastra). Rabbit anti-goat linker antibody (Agilent/DAKO, Santa Clara, CA, USA) was used for primary goat antibodies. Each IHC stain was performed using a 4-channel TECAN Freedom Evo liquid handling platform (Männedorf, Switzerland).

To determine the TFH-cell phenotype, we used 7 TFH cell-related markers, including CXCR5, CD134, PD1, BCL6, CXCL13, CD10, and ICOS. The primary antibodies used are listed in Table 3. To increase sensitivity and specificity, 3 anti-CXCR5 antibodies were tested for the study. Two of them, mouse anti-CXCR5 monoclonal antibody 51505 (Bio-Techne/R&D Systems, Minneapolis, MN, USA) and a rabbit anti-CXCR5 polyclonal antibody (ThermoFisher/Invitrogen, Waltham, MA, USA), showed an equivalent and specific IHC staining pattern. These two well-validated antibodies were used in the study. The third antibody evaluated (rabbit anti-CXCR5 recombinant monoclonal antibody JB11-40, Bio-Techne/NovusBio) showed nonspecific staining in paraffin section IHC and was excluded from further testing. Each TFH cell marker antibody was optimized using tissue microarrays and whole tissue sections of hyperplastic lymph nodes and palatine tonsil tissue.

The TFH cell phenotype of a PTCL case was determined if at least 4 of the 7 TFH cell markers in the abnormal T cells were positive in addition to CD4. A case was classified as CXCR5+ if the abnormal T cells showed specific CXCR5 immunostaining. A case was declared CXCR5-negative if the abnormal T cells showed negative immunostaining with both CXCR5 antibodies used. Sensitivity, specificity, and accuracy were calculated based on the immunohistochemical findings.

The Mann-Whitney U test was used to correlate the expression of CXCR5 in the TFHL and non-TFH PTCL cases.

### 4.3. Multiple Fluorescence Antigen Immunostaining

Simultaneous multiple-fluorescence antigen immunostaining was performed on paraffin sections. Following appropriate pretreatment, the slides were treated with a cocktail of two or three primary antibodies of mouse, rabbit, and goat origin (Table 3). This step was followed by a single-step incubation with a secondary antibody cocktail of highly cross-absorbed donkey anti-mouse, anti-rabbit, and anti-goat antibodies labeled with Alexa Fluor Plus 647, Alexa Fluor Plus 488, and Alexa Fluor Plus 555, respectively (all from ThemoFisher/Invitrogen). ProLong Glass Antifade with NucBlue Stain (ThemoFisher/Invitrogen) was used to mount the slides.

### 4.4. EBER In Situ Hybridization

The presence of Epstein-Barr virus (EBV) was detected by EBER in situ hybridization using a blend of EBER1 (5′FAM-TCACCACCCGGGACTTGTACCCGGGACGGG) and EBER2 (5′FAM-TCCTCCCCCGGGACTTGACCTCGGGTCGG) custom-made FITC-labeled oligonucleotide probes. An anti-FITC mouse monoclonal antibody (Thermofisher/Invitrogen) was utilized to detect the hybridized probe.

## 5. Conclusions

Here, we have shown that CXCR5 is a defining marker of TFHLs. Since CXCR5 plays a central role in the establishment of follicles and the maintenance of the follicular microenvironment, our results suggest that the presence of CXCR5 in the neoplastic TFH cells may direct their distribution within the lymph nodes and influence the development of the pathological FDC network. The evaluation of the distribution of neoplastic TFH cells helped us refine the morphological criteria of TFHL subtypes. According to our observations, the main difference between TFHL-AI, TFHL-F, and TFHL-NOS was that the formers always showed some degree of FDC alterations and intrafollicular neoplastic cells, while the latter either completely lacked follicles or showed no intrafollicular neoplastic TFH cells. TFHL-AI-1, TFHL-F, and TFHL-AI-3 cases showed dominant intrafollicular neoplastic cell accumulation, whereas we found both intrafollicular and extrafollicular dominant cases in the TFHL-AI-2 cohort. The most distinctive difference between the TFHL-AI-2 and TFHL-AI-3 cases was the structure of the FDC network. The TFHL-AI-2 cases showed atrophic or minimally expanded irregular FDC networks, while the TFHL-AI-3 cases were characterized by marked FDC expansion and diffusely confluent FDC networks. These features may be helpful in the specific diagnosis of TFHL types, even in core biopsy specimens. Further studies are needed to identify other factors influencing the migration of neoplastic TFH cells in lymph nodes affected by TFHL.

## Figures and Tables

**Table 1:** Sensitivity, specificity, and accuracy of the TFH cell markers studied.

	Sensitivity	Specificity	Accuracy
CXCR5	85% (67/79) (* 98% (62/63))	0.91	0.86
PD1	99% (77/78)	0.73	0.96
BCL6	94% (67/71)	1	0.95
CD134	94% (74/79)	0.60	0.90
CXCL13	87% (69/79)	0.91	0.88
ICOS	83% (65/78)	0.70	0.82
CD10	63% (50/79)	1	0.67

* With the exclusion of TFHL-NOS cases.

**Table 2:** Summary of morphological characteristics of TFHL cases.

	TFHL-AI-1	TFHL-AI-2	TFHL-AI-3	TFHL-F	TFHL-NOS
**Architectural alterations**	Partial disarrangement; Follicular hyperplasia-like features	Partial or complete disarrangement in the LN architecture	Complete diffuse effacement of the LN architecture	Partial disarrangement; Follicle-like nodules mainly composed of abnormal T cells.	Type A, diffuse infiltrate of medium to large neoplastic cells; Type B, T-zone lymphoma pattern; Type C, polymorphic infiltrate without follicles
**Extrafollicular/paracortical compartment**	Minimal paracortical alterations; HEVs may penetrate radially into GC; Scarce CD30+ (EBV+/−) large B-cells/HRS-like cells	Polymorphic infiltrate with HEV proliferation; CD30+ (EBV+/−) large B/HRS-like cells can be present	Polymorphic infiltrate with arborizing HEVs. CD30+ (EBV+/−) large BB/HRS-like cells often present	Disrupted paracortical tissue with mild HEV proliferation	Type A, diffuse infiltrate; Type B & C, polymorphic infiltrate with HEV proliferation; CD30+ (EBV+/−) large B cells/RS-like cells can be present
**Follicles/GC B cells**	Largely retained GC B cell population; CD30+ (EBV+/−) intrafollicular large B/HRS-like cells can be present	Depleted follicles, scarce GC B cells	GC B cells absent	No residual GC B cells; CD30+ (EBV+/−) intrafollicular large B/HRS-like cells can be present	Type A & C, no follicles; Type B, atrophic follicles with residual GC B cells
**Mantle zone/small B-cell aggregates**	Distorted mantle zones; Displaced small B-cell aggregates can be present	Displaced mantle zone-type small B cells pushed to the periphery of the LN	Haphazardly distributed sheets of small B cells adjacent to the FDC meshwork or confined to the periphery of the LN	Sheets of small B cells adjacent to the neoplastic T-cell nodules or pushed to the subcapsular region	Type A, few small B cells; Type B, almost intact mantle zones; Type C, sheets of mantle zone-type small B-cells
**FDC meshwork**	Vanishing polarization; marginally expanded FDC meshwork	Dense, partly concentric, focally expanded, focally interconnected	Dense, markedly expanded, and widely interconnected; HEVs can be entrapped	Dense, irregular, marginally expanded, focally confluent FDC meshwork	Type A: no FDCs; Type B: atrophic FDC meshwork; Type C: absent or delicate traces of FDCs
**Neoplastic cells**	Small to medium abnormal TFH cells with pale cytoplasm	Medium to large abnormal TFH cells often with pale cytoplasm	Medium to large abnormal TFH cells with pale cytoplasm	Small to medium abnormal TFH cells sometimes with pale cytoplasm	Type A, medium to large abnormal T cells with pale cytoplasm; Type B, medium-sized abnormal T cells; Type C, Medium to large abnormal T cells with pale cytoplasm
TFH phenotype */localization of neoplastic T cells	Complete/Accumulated at the boundary of GCs, minimal paracortical population	Complete/Type A, mainly intra/perifollicular; Type B, mainly paracortical	Complete/Intrafollicular (within FDC meshwork) > paracortical	Complete/Dense intrafollicular aggregates and scattered extrafollicular	Type A, CXCR5+/CD10-; Type B, CXCR5-/CD10-; Type C, CXCR5-/CD10- > CXCR5+/CD10+; Exclusively paracortical localization

EBV, Epstein–Barr virus; FDC, follicular dendritic cell; GC, germinal center; HEV, high endothelial venule; HRS, Hodgkin–Reed–Sternberg; TFH, follicular T helper; TFHL, TFH cell lymphoma; TFHL-AI-1, -AI-2, -AI3, TFHL angioimmunoblastic-type, pattern 1, 2, and 3; TFHL-F, TFHL follicular-type; TFHL-NOS, TFHL not otherwise specified-type. * Complete—positive for at least 4/7 TFH markers, including CXCR5.

**Table 3:** List of antibodies used in the study.

Antibody/Clone	Origin	Source
BCL6/LN22	Mouse	Leica/Novocastra
CD3/H-12	Mouse	Santa Cruz
CD4/N1GU0	Mouse	Thermofisher/eBioscience
CD8/C8/144B	Mouse	Santa Cruz
CD10/56C6	Mouse	Leica
CD20/L26	Mouse	Leica
CD21/ER3093	Rabbit	Histopathology *
CD23/1B12	Mouse	Leica
CD30/JCM182	Mouse	Leica
CD134/Ber-Act35	Mouse	Santa Cruz
CXCL13/polyclonal	Goat	Bio-Techne/R&D Systems
CXCR5/polyclonal	Rabbit	Atlas Antibodies
CXCR5/51505	Mouse	Bio-Techne/R&D Systems
EBV-LMP1/CS1-4	Mouse	Agilent/DAKO
ICOS/polyclonal	Goat	Bio-Techne/R&D Systems
IgD/polyclonal	Rabbit	Agilent/DAKO
MEF2B/polyclonal	Rabbit	Atlas Antibodies
PD1/polyclonal	Goat	Bio-Techne/R&D Systems
TIA-1/2G9A10F5	Mouse	Beckman-Coulter

* Histopathology Ltd., Pecs, Hungary.

## Data Availability

The data presented in this study are available on request from the corresponding author. The data are not publicly available due to ethical restrictions.

## Supplementary Materials

The following supporting information can be downloaded at: https://www.mdpi.com/article/10.3390/ijms25010428/s1.

## Abbreviations

PTCL	Peripheral T-cell lymphoma
TFH	Follicular T helper
TFHL	Follicular T helper cell lymphoma
TFHL-AI	Follicular T helper cell lymphoma angioimmunoblastic-type
TFHL-AI-1	Follicular T helper cell lymphoma angioimmunoblastic-type, pattern 1
TFHL-AI-2	Follicular T helper cell lymphoma angioimmunoblastic-type, pattern 2
TFHL-AI	Follicular T helper cell lymphoma angioimmunoblastic-type, pattern 3
TFHL-F	Follicular T helper cell lymphoma follicular-type
TFHL-NOS	Follicular T helper cell lymphoma not otherwise specified-type
CXCR5	CXC chemokine receptor type 5
CXCL13	CXC chemokine ligand 13
BCL6	B-cell lymphoma 6
PD1	Programmed cell death protein 1
ICOS	Inducible T-cell costimulator
MEF2B	Myocyte enhancer binding factor 2 B
EBV	Epstein-Barr virus
EBER	EBV-encoded small RNA
LMP1	EBV-encoded latent membrane protein 1
DLBCL	Diffuse large B-cell lymphoma
TCR	T-cell receptor
GC	Germinal center
B-IBs	B immunoblasts
HRS-like	Hodgkin-Reed-Sternberg-like
FDC	Follicular dendritic cell
HEV	High endothelial venule
IHC	Immunohistochemistry
